# Psychometric validation of the Turkish Brief General AI Self-Efficacy Scale: examining its role in AI anxiety, acceptance, and demographic predictors

**DOI:** 10.3389/fpsyg.2026.1714147

**Published:** 2026-02-04

**Authors:** Sedef Çelik Demirci, Metin Besalti, Ümit Kul

**Affiliations:** 1 Department of Mathematics Education, Faculty of Education, Artvin Coruh University, Artvin, Türkiye; 2 Department of Educational Sciences, Faculty of Education, Artvin Coruh University, Artvin, Türkiye; 3 Department of Educational & Psychological Studies, Faculty of Education, University of South Florida, Tampa, FL, United States

**Keywords:** AI anxiety, AI self-efficacy, artificial intelligence, generative AI acceptance, pre-service teachers, scale adaptation

## Abstract

**Background:**

The rapid integration of artificial intelligence (AI) technologies in education highlights the urgency of understanding pre-service teachers’ readiness to adopt these tools effectively. Although prior research has separately examined AI self-efficacy, AI anxiety, and generative AI acceptance, few studies have investigated their interrelations within a unified framework. This study linguistically adapted and psychometrically validated the Turkish version of the Brief General AI Self-Efficacy Scale (GSE-6AIS) and explored its associations with AI anxiety, generative AI acceptance, and demographic characteristics.

**Methods:**

Data were collected from 941 pre-service teachers (52.4% female, 47.6% male; *M* = 21.97 years, SD = 1.45) recruited via convenience sampling from seven Turkish universities. Confirmatory factor analyses supported the scale’s unidimensional structure and internal consistency, and multi-group analyses indicated gender invariance.

**Results:**

The results showed that higher AI knowledge predicted greater self-efficacy and generative AI acceptance, and lower AI anxiety, whereas gender and computer use showed no significant effects. Mediation analyses revealed that AI self-efficacy partially mediated the relationship between AI anxiety and generative AI acceptance, highlighting its role as a key psychological mechanism. Integrating Bandura’s self-efficacy theory with the Technology Acceptance Model (TAM), findings highlight AI self-efficacy as a central mechanism linking anxiety to generative AI acceptance.

**Conclusion:**

These findings indicate that the Turkish AI Self-Efficacy Scale is a reliable and valid measure and underscore the importance of fostering self-efficacy to reduce anxiety and enhance acceptance of generative AI in educational contexts. The results have practical implications for teacher education programs aiming to prepare future educators for the increasing presence of AI in learning environments.

## 1 Introduction

Artificial intelligence (AI) has played a transformative role in technological advancements in recent years and has become a central force in education, healthcare, economics, and various other fields. As one of the most important elements of modern technology, AI is particularly reshaping higher education by influencing both teaching and learning processes and reshaping students’ academic experiences (Bates et al., 2020; Crompton and Burke, 2023). University students are increasingly integrating AI-based tools into their learning processes to improve their daily academic tasks (Chan and Hu, 2023). These tools are becoming more embedded in students’ learning experiences, with usage rates growing each year. According to a report by the Digital Education Council, a large number of students (86%) actively incorporate these tools into their education (Digital Education Council, 2024).

AI is now an essential part of higher education, supporting everything from learning platforms and automated assignment tools to translation services and data analysis programs. For example, AI-based learning management systems enhance the academic experience of learners by adjusting the content based on each student’s pace and individual needs (Adiguzel et al., 2023). Furthermore, generative AI tools help students with tasks such as writing assignments and solving complex problems. AI translation tools facilitate access to multilingual resources, effectively overcoming language barriers (Bicknell et al., 2023; Kohnke et al., 2023). Additionally, data analysis applications significantly contribute to research-driven projects by enabling students to process and interpret large datasets, thereby enhancing their scientific studies (Chen et al., 2020). The increasing use of these technologies indicates that students are gradually integrating AI into their daily academic activities as a fundamental tool.

Although university students widely use AI and significantly improve their learning processes, it also brings up some ethical concerns, such as academic integrity violations. Recent research from Hong Kong demonstrates that social contagion amplifies AI adoption, with lower-GPA students reporting more permissive views toward illicit use, while policy awareness and perceived detection effectiveness promote ethical, responsible integration (Lo and Chan, 2025). Additionally, it poses pedagogical challenges, including a potential decline in problem-solving and critical thinking skills, along with social issues related to digital inequality and data privacy (Kasneci et al., 2023; Nguyen et al., 2023; Ouyang et al., 2022; Zhang et al., 2024). As the educational integration of AI accelerates, its impact extends beyond technological adoption to encompass learners’ psychological responses, particularly AI anxiety and AI self-efficacy, which jointly shape their acceptance and engagement with generative AI tools (Wang and Chuang, 2024; Wang and Wang, 2022).

### 1.1 Theoretical framework

#### 1.1.1 Self-efficacy as a foundational mechanism

Self-efficacy plays a critical role in shaping individuals’ personal beliefs and behaviors (Compeau and Higgins, 1995). According to Bandura’s (1977) social cognitive theory (SCT), self-efficacy represents individuals’ overall beliefs in their ability to effectively manage and overcome challenging circumstances. In technology contexts, self-efficacy plays a pivotal role in the adoption and effective use of new technologies (Zimmerman, 2000). With the rapid development of AI technologies, students’ self-efficacy regarding AI has become an increasingly relevant issue. In AI contexts, AI self-efficacy is defined as the belief in one’s own capability to proficiently utilize AI tools, as well as the perception of their own skillfulness in doing so (Morales-García et al., 2024; Wang and Chuang, 2024). Individuals with high self-efficacy often have more optimistic views about the ease of use and advantages of technology, which makes them more likely to adopt these tools successfully. For instance, AI-powered learning platforms in education allow students to personalize their learning experiences, and research indicates that students with higher AI self-efficacy are better at using these platforms effectively (Chen et al., 2024; Liang et al., 2023).

The Technology Acceptance Model (TAM) (Davis, 1989) identifies perceived usefulness and perceived ease of use as primary determinants of technology adoption. Compeau and Higgins (1995) extended this model by demonstrating that computer self-efficacy significantly predicts both perceived ease of use and actual usage behavior. Building on this foundation, TAM3 (Venkatesh and Bala, 2008) introduced self-efficacy as an anchor variable that shapes users’ perceptions of ease of use through their judgments of personal capability. This version of the model emphasizes the importance of addressing psychological antecedents, such as self-efficacy and anxiety, as a means of indirectly improving technology acceptance by enhancing perceptions of usefulness and ease of use. While TAM primarily focuses on immediate cognitive determinants of technology adoption, the present study shifts attention to more distal psychological factors, specifically self-efficacy and anxiety, which influence acceptance in accordance with the hierarchical structure proposed in TAM3. Similarly, the Unified Theory of Acceptance and Use of Technology (UTAUT) framework identifies effort expectancy and performance expectancy as key predictors of behavioral intention, both of which are influenced by users’ confidence in their technological capabilities (Venkatesh et al., 2003). These models collectively suggest that AI self-efficacy serves as a foundational psychological mechanism that indirectly influences technology acceptance by shaping users’ perceptions of ease and utility.

Empirical studies support these linkages. In the context of generative AI, Liang et al. (2023) found that interactions with such technologies enhance learning outcomes through self-efficacy, while Yilmaz and Yilmaz (2023) experimentally confirmed that using ChatGPT improves programming self-efficacy. Hong’s (2022) study revealed a positive correlation between AI self-efficacy and both AI acceptance and the intention to use AI. Similarly, Wang and Chuang (2024) demonstrated that AI self-efficacy scales reinforce the broader acceptance of these technologies. These findings emphasize that self-efficacy both directly and indirectly aids in the effective use and adoption of AI technologies, while also enhancing personalized learning experiences in education. Furthermore, as AI usage strengthens self-efficacy, this creates a reinforcing cycle of increased confidence and engagement.

#### 1.1.2 AI anxiety as a barrier to technology adoption

While self-efficacy fosters confidence and acceptance, psychological barriers such as AI anxiety can hinder individuals’ willingness to engage with AI technologies. AI anxiety refers to individuals’ feelings of concern, fear, or discomfort regarding AI technologies, and it significantly influences the adoption of these technologies (Johnson and Verdicchio, 2017; Li and Huang, 2020; Wang and Wang, 2022). Within the framework of Transactional Stress Theory (Lazarus and Folkman, 1984), AI anxiety represents a primary threat appraisal involving perceived risks to autonomy, control, or professional identity. When individuals evaluate their coping resources, such as self-efficacy, as insufficient, avoidance responses become more likely. From the perspective of TAM3, anxiety functions as a psychological antecedent that heightens cognitive load and diminishes perceived behavioral control, thereby reducing acceptance (Venkatesh and Davis, 2000; Venkatesh and Bala, 2008). Similarly, in the UTAUT model, anxiety weakens effort expectancy and consequently lowers the intention to adopt AI technologies (Venkatesh et al., 2003).

Previous research has examined both the sources and consequences of AI anxiety. For instance, Johnson and Verdicchio (2017) suggest that AI anxiety often stems from concerns about technological autonomy and loss of control, while Stănescu and Romașcanu (2024) found that such anxiety reduces individuals’ attitudes toward AI and their willingness to use it. Kaya et al. (2024) conducted a study with 350 university students to examine the impact of AI anxiety on students’ attitudes and found a negative relationship between self-efficacy and anxiety. Their findings suggested that individuals with high levels of self-efficacy may be more resistant to the negative effects of AI anxiety, which in turn could positively influence their attitudes toward technology. Similarly, Chen et al. (2024) investigated the psychological factors influencing the use of AI technologies among L2 learners. The results indicated that AI self-efficacy positively affects learners’ attitudes toward AI and their practical application of these technologies. The study also highlighted that individuals with higher self-efficacy experience lower levels of AI anxiety, as their confidence in using these tools helps mitigate anxiety.

Avcı (2024) investigated the acceptance of generative AI technologies among university students, considering the roles of demographic characteristics, creative mindsets, AI anxiety, and attitudes. The study found that positive attitudes toward AI significantly influence GAIAS. While AI anxiety and negative attitudes were examined, the study found that positive attitudes and growth, creative mindsets were more critical determinants. Similarly, Ayanwale et al. (2024) explored pre-service teachers’ engagement with learning AI, focusing on the impact of anxiety toward AI. They found that anxiety toward AI can inversely affect student engagement in AI technologies. Another study done by Wang et al. (2024) investigated the factors influencing students’ intentions to learn AI, focusing on the roles of AI learning anxiety, learning motivation, and self-efficacy. They found that AI anxiety negatively affects students’ learning motivation, self-efficacy, and intentions to learn AI. Schiavo et al. (2024) explored the relationship between AI literacy, anxiety, and acceptance based on the TAM model using a survey of 313 respondents. The findings revealed that AI literacy positively influences AI acceptance, while anxiety has a minimal direct negative effect but plays a significant mediating role. These findings demonstrate that AI anxiety, self-efficacy, and the acceptance of generative AI are interconnected in complex ways, with individual differences playing a critical role in this dynamic.

#### 1.1.3 Mediating role of AI self-efficacy between AI anxiety and acceptance

The influence of AI anxiety on GAIAS can be conceptualized through a dual-pathway framework integrating SCT, TAM/TAM3, UTAUT, and transactional stress theory. On one hand, AI anxiety exerts a direct negative effect on acceptance by increasing perceived risk and avoidance motivation (Johnson and Verdicchio, 2017; Venkatesh and Davis, 2000). On the other hand, AI anxiety also affects acceptance indirectly via AI self-efficacy. Within the transactional stress framework, self-efficacy serves as a secondary appraisal of coping resources, enabling individuals to reframe perceived threats as manageable challenges (Lazarus and Folkman, 1984; Bandura, 1997). Higher AI self-efficacy strengthens perceptions of ease of use (TAM) and effort expectancy (UTAUT), which in turn promote technology acceptance (Venkatesh and Bala, 2008; Venkatesh et al., 2003, 2012).

Empirical evidence supports this dual-pathway mechanism. Interactions with generative AI tools enhance learning outcomes by increasing self-efficacy (Liang et al., 2023), and experimental use of ChatGPT has been shown to improve programming self-efficacy (Yilmaz and Yilmaz, 2023). AI self-efficacy is positively associated with both acceptance and intention to use AI technologies (Hong, 2022; Wang and Chuang, 2024), whereas AI anxiety predicts lower acceptance and engagement (Schiavo et al., 2024; Stănescu and Romașcanu, 2024). Similarly, Tekin (2024) demonstrated that incorporating AI self-efficacy and AI anxiety into the TAM substantially improves the prediction of teachers’ AI adoption. AI anxiety was found to diminish perceived usefulness and ease of use, whereas self-efficacy strengthened intention to use AI.

A similar pattern emerges in recent work, indicating that AI literacy and attitudes toward AI mediate the association between AI anxiety and GAIAS among university students, further illustrating the complex processes through which anxiety shapes technology-related perceptions and behaviors (Cengiz and Peker, 2025). Given that anxiety can trigger avoidance independently of efficacy beliefs, AI self-efficacy is expected to partially mediate the relationship between AI anxiety and GAIAS. This expectation aligns with prior findings showing that cognitive factors such as self-efficacy and AI literacy explain only part of anxiety’s influence on technology adoption (Schiavo et al., 2024; Wang et al., 2024).

#### 1.1.4 Demographic influences on AI-related constructs

Individual differences in AI self-efficacy, anxiety, and acceptance may also be influenced by demographic characteristics, though findings are mixed. Gender effects have been inconsistent. Zhang et al. (2023) found that gender moderated TAM relationships among German pre-service teachers, with stronger effects of perceived usefulness and subjective norm for females and greater influence of perceived ease of use for males. However, Chen et al. (2024) and Kaya et al. (2024) found no gender differences in AI self-efficacy or anxiety among Chinese second language learners and Turkish/United Kingdom university students, respectively. These inconsistencies may reflect cultural variations, sample characteristics, or the novelty of generative AI technologies.

Beyond gender, domain-specific knowledge and general technology proficiency show more consistent effects. AI knowledge is predicted to be a strong predictor based on Bandura’s (1997, p. 80) mastery experience principle: “The most effective way of creating a strong sense of efficacy is through mastery experiences.” Individuals with greater AI knowledge possess more successful prior interactions with AI tools, which should strengthen efficacy beliefs and reduce anxiety through familiarization. Schiavo et al. (2024) provided direct empirical support, demonstrating that AI literacy positively predicted acceptance and negatively predicted anxiety. Computer use frequency is predicted to positively influence AI-related constructs through skill transfer mechanisms. Wang and Chuang (2024) argued that general technology proficiency provides a foundation for AI-specific confidence. In the Turkish context, Terzi (2020) adapted the Artificial Intelligence Anxiety Scale and reported moderate anxiety levels among university students, while Avcı (2024) found that AI knowledge significantly predicted GAIAS, though gender showed only a small positive correlation.

### 1.2 The present study and hypotheses development

Despite the exponential growth of AI in educational settings, there remains a significant gap in understanding the multifaceted relationship between AI anxiety, AI self-efficacy, and GAIAS, particularly in educational contexts. Existing research has primarily examined these constructs in isolation, with limited integration of theoretical frameworks such as SCT, TAM/TAM3, UTAUT, and transactional stress theory. Empirical studies on generative AI adoption remain scarce, and the mediating role of AI self-efficacy has rarely been tested explicitly. Furthermore, cultural and contextual variations in the relationships among AI anxiety, AI self-efficacy, and GAIAS are largely unexplored. The current study addresses this knowledge gap by adapting the General AI Self-Efficacy Scale to Turkish and examining the relationships among these three critical constructs, alongside relevant demographic variables, among pre-service teachers.

While previous research has examined these variables individually (Avcı, 2024; Wang et al., 2024), the present study offers a comprehensive investigation of their interrelationships through an integrated theoretical framework combining SCT, TAM/TAM3, UTAUT, and transactional stress theory. Prior research, such as Schiavo et al. (2024) in an Italian sample, demonstrates that anxiety significantly influences AI acceptance; however, the specific mediating role of AI self-efficacy in this process remains underexplored. By focusing on Turkish pre-service teachers, this study addresses the limited literature on AI acceptance in non-Western educational contexts and enables cross-cultural comparisons with recent findings from German (Zhang et al., 2023) and Chinese (Chen et al., 2024) teacher samples.

Based on this integrated theoretical framework, the following hypotheses are proposed and tested.

*Hypothesis 1 (H1)*: The Turkish version of the General AI Self-Efficacy Scale (GSE-6AIS) is expected to demonstrate a unidimensional factor structure with high internal consistency.

The original GSE-6AIS (Morales-García et al., 2024) exhibited a robust unidimensional structure and excellent internal consistency (*α* = 0.91) among Peruvian university students. Cross-cultural adaptation research (Beaton et al., 2000) indicates that when conceptual equivalence is maintained through rigorous translation protocols, factor structures typically remain stable across linguistically similar populations. Given the universal nature of self-efficacy as a psychological construct (Bandura, 1997) and the successful adaptation of AI-related scales in Turkish contexts (Terzi, 2020), the Turkish GSE-6AIS is expected to replicate this structure.

*Hypothesis 2a (H2a)*: AI anxiety (AIAS) will be significantly negatively correlated with AI self-efficacy (GSE-6AIS).

AI anxiety is expected to be significantly negatively correlated with AI self-efficacy. According to Bandura’s (1977) SCT, emotional arousal, including anxiety, serves as one of four sources of efficacy information. Physiological and affective states are interpreted as indicators of personal competence, so heightened anxiety tends to weaken efficacy beliefs through negative self-evaluation. Empirical evidence supports this inverse relationship. Kaya et al. (2024) found negative correlations between AI anxiety and self-efficacy among Turkish university students, and Chen et al. (2024) reported similar patterns among Chinese learners. When individuals experience anxiety about AI’s unpredictability or potential for job displacement (Wang and Wang, 2022), their confidence in mastering AI tools diminishes.

*Hypothesis 2b (H2b)*: AI anxiety (AIAS) will be significantly negatively correlated with GAIAS.

AI anxiety is expected to be significantly negatively correlated with GAIAS. TAMs consistently show that affective barriers reduce adoption intentions. In TAM (Venkatesh and Davis, 2000), anxiety is conceptualized as an inhibitor that increases perceived behavioral control costs. Johnson and Verdicchio (2017) describe AI anxiety as an avoidance motivation rooted in concerns about technological autonomy. Empirical evidence supports this view. Schiavo et al. (2024) found that AI anxiety had negative effects on acceptance among Italian adults, and Stănescu and Romașcanu (2024) reported that anxiety reduced attitudes toward AI and willingness to use AI technologies. In the Turkish context, Avcı (2024) found that anxiety negatively influenced GAIAS among university students.

*Hypothesis 2c (H2c)*: AI self-efficacy (GSE-6AIS) will be significantly positively correlated with GAIAS.

AI self-efficacy is expected to be significantly positively correlated with GAIAS. This hypothesis follows directly from the TAM (Davis, 1989) and its extensions. Compeau and Higgins (1995) demonstrated that computer self-efficacy predicts both perceived ease of use and actual computer usage, establishing self-efficacy as a critical antecedent to technology acceptance. In the AI domain, Hong (2022) found positive correlations between AI self-efficacy and both AI acceptance and intention to use AI among Korean users. Wang and Chuang (2024) showed that AI self-efficacy predicted broader acceptance of AI technologies among Taiwanese university students. Liang et al. (2023) reported that student interactions with generative AI enhanced learning outcomes through self-efficacy, suggesting a reinforcing cycle.

*Hypothesis 3 (H3)*: Demographic characteristics, specifically gender, self-reported level of AI knowledge, and frequency of computer use, will significantly predict AI self-efficacy (GSE-6AIS), AI anxiety (AIAS), and GAIAS.

Demographic characteristics, specifically gender, self-reported level of AI knowledge, and frequency of computer use, are expected to significantly predict AI self-efficacy, AI anxiety, and GAIAS. This hypothesis draws on multiple theoretical perspectives on individual differences in technology adoption. Gender effects have been suggested by research documenting gaps in STEM self-efficacy and technology usage (Venkatesh and Morris, 2000), although recent findings are inconsistent. AI knowledge is predicted to be a strong positive predictor based on Bandura’s (1997) mastery experience principle, which states that successful prior interactions with technology strengthen efficacy beliefs and reduce anxiety through familiarization. Empirical evidence supports this expectation. Schiavo et al. (2024) found that AI literacy positively predicted acceptance and negatively predicted anxiety. Frequency of computer use is also expected to positively influence AI-related constructs through skill transfer mechanisms, as general technology proficiency provides a foundation for AI-specific confidence (Wang and Chuang, 2024).

*Hypothesis 4 (H4)*: AI self-efficacy (GSE-6AIS) will partially mediate the relationship between AI anxiety (AIAS) and GAIAS.

AI self-efficacy is expected to partially mediate the relationship between AI anxiety and GAIAS. This hypothesis integrates SCT (Bandura, 1977, 1997), transactional stress theory (Lazarus and Folkman, 1984), with TAM3 (Venkatesh and Bala, 2008). In the transactional model, stress responses unfold in two stages: primary appraisal, which evaluates whether AI poses a threat, and secondary appraisal, which evaluates coping resources such as self-efficacy. When individuals appraise AI as threatening but perceive strong coping resources, the threat is cognitively reappraised as manageable, enabling acceptance rather than avoidance. This process reflects the indirect effect of AI anxiety on GAIAS through AI self-efficacy.

However, partial rather than full mediation is expected. Johnson and Verdicchio (2017) argue that AI anxiety reflects existential concerns about human agency and autonomy that may directly trigger avoidance independently of efficacy beliefs. Pre-service teachers may also harbor anxieties rooted in professional identity threats, ethical concerns, or philosophical objections that persist regardless of confidence in personal capability. TAM3 suggests that anxiety operates through multiple pathways, including cognitive (via self-efficacy) and motivational (via intrinsic motivation). Prior empirical work supports partial mediation: Schiavo et al. (2024) found that AI literacy mediated 38 percent of anxiety’s total effect on acceptance, while Wang et al. (2024) reported that self-efficacy mediated 22 percent of anxiety’s effect on learning intentions.

**Figure 1:**
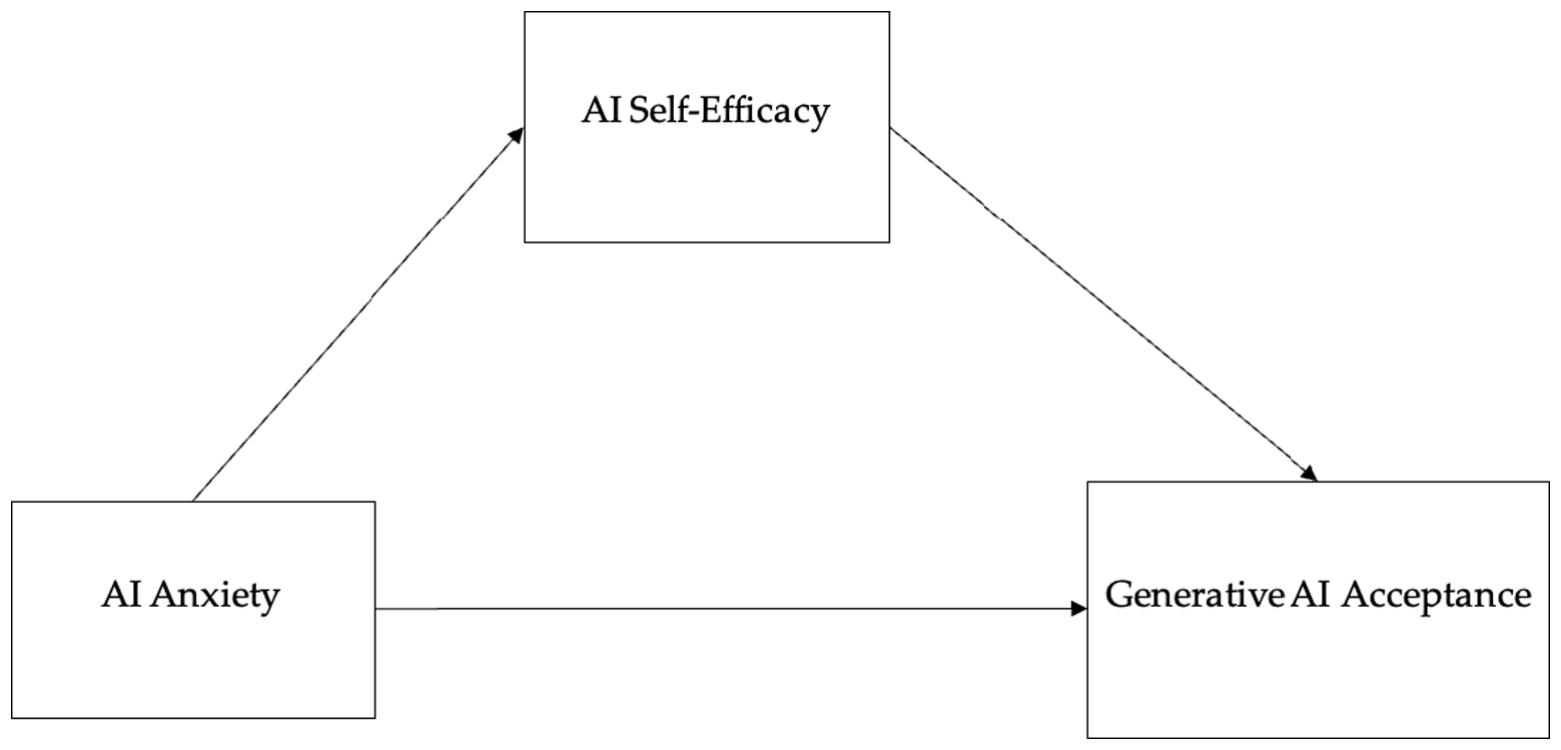
Hypothesized mediation model.

Figure 1 presents the hypothesized model for this study. The model integrates Bandura’s (1977, 1997) SCT, TAM (Davis, 1989; Venkatesh and Bala, 2008), and transactional stress theory (Lazarus and Folkman, 1984). AI anxiety is hypothesized to negatively influence both AI self-efficacy and GAIAS. AI self-efficacy is proposed to mediate the anxiety-acceptance relationship while also exerting a direct positive effect on acceptance. Demographic variables, including AI knowledge, computer use, and gender, serve as background factors influencing all three core constructs. By systematically testing these hypotheses among Turkish pre-service teachers, this study advances theoretical understanding of psychological mechanisms underlying AI technology adoption and provides practical guidance for designing teacher education interventions that address both affective (anxiety) and cognitive (self-efficacy) barriers.

## 2 Methods

### 2.1 Participants

An initial sample of 969 pre-service teachers enrolled in teacher education programs at seven universities across Türkiye participated. In line with a prespecified screening rule, 28 cases with more than 3% missing responses were excluded, yielding a full analytic sample of 941 participants used for psychometric validation. The gender distribution was balanced (52.4% female, *n* = 493; 47.6% male, *n* = 448). Participants ranged in age from 19 to 26 years (*M* = 21.97, SD = 1.45). Recruitment was conducted via convenience sampling across multiple regions to enhance contextual diversity; however, this approach may limit generalizability beyond similar teacher-education populations (Ary et al., 2014; Creswell, 2014). Detailed distributions of self-reported AI knowledge and computer use are presented in Table 1. Inclusion criteria were current enrolment in a teacher education program and provision of informed consent. Exclusion criteria included >3% missing responses, duplicate submissions, and indications of inattentive responding (e.g., unrealistically short completion times). Participation was voluntary and uncompensated.

**Table 1:** Demographic characteristics of the participants (*N* = 941).

Characteristic	Category	*n*	%
Gender	Female	493	52.4
	Male	448	47.6
Level of knowledge about AI	No knowledge	136	14.5
	Basic knowledge	564	59.9
	Moderate knowledge	176	18.7
	In-depth knowledge	65	6.9
Level of computer use	Minimal user	4	0.4
	Below-average user	402	42.7
	Average user	234	24.9
	Expert user	301	32.0

Age range: 19–26 years; Mean age = 21.97 years (SD = 1.45).

### 2.2 Procedure

Data were collected via an anonymous online survey between October and December 2024. Ethical approval was obtained from the University Scientific Research and Ethical Review Board (Approval no: E-18457941-050.99-135153; September 2024). Participation was voluntary, and informed consent was obtained electronically prior to survey access.

For linguistic adaptation, the GSE-6AIS underwent forward–backward translation by bilingual experts, expert panel review (AI and psychometrics), and cognitive pretesting with 12 pre-service teachers. Pilot responses were used only to refine wording and were not included in the final analyses. To address the methodological constraints of validation studies, we adopted a split-sample validation approach (Brown, 2015). The total sample was randomly divided to perform EFA and CFA on independent subsamples, ensuring that the factor structure was both discovered and verified without overfitting.

An *a priori* power analysis in G*Power 3.1 (Faul et al., 2009) for multiple linear regression with three predictors (gender, AI knowledge, computer use), assuming a medium effect size of *f*^2^ = 0.15, *α* = 0.05, and power (1 − *β*) = 0.95, indicated a minimum required sample of *N* = 119. For confirmatory factor analysis/SEM with six ordinal indicators (GSE-6AIS items) estimated via WLSMV, our sample (*N* = 941) far exceeds commonly cited adequacy guidelines (e.g., *N* > 200; Kline, 2010) and provides ample power for multi-group invariance testing by gender.

### 2.3 Instruments

#### 2.3.1 Demographic Questionnaire

A brief survey collected age, gender, self-reported level of AI knowledge (1 = No knowledge to 4 = In-depth knowledge), and level of computer use (1 = Minimal user to 4 = Expert user). These variables were analyzed as predictors and correlates of the focal constructs.

#### 2.3.2 Brief General AI Self-Efficacy Scale (GSE-6AIS)

The GSE-6AIS (Morales-García et al., 2024) comprises 6 items assessing confidence in effectively using AI tools. Response options are on a 4-point Likert scale (1 = Not at all true, 4 = Exactly true), treated as ordered categorical. Higher scores indicate greater AI self-efficacy. Example items include “No matter what comes my way, I’m usually able to handle it with the support of AI.” and “I am confident that I could deal efficiently with unexpected events involving AI tools.” The Turkish adaptation in this study followed standard cross-cultural procedures (forward-back translation, expert review, pilot testing; see Procedure; Beaton et al., 2000). In the original study, internal consistency was high (*α* = 0.91). In the present sample, internal consistency was good (*α* = 0.85; *ω* = 0.86). The mean score of the 6 items was used as the composite score.

#### 2.3.3 Artificial Intelligence Anxiety Scale (AIAS)

The AIAS (Wang and Wang, 2022; Turkish adaptation by Terzi, 2020) includes 21 items across four domains: Learning Anxiety, Job Replacement Anxiety, Sociotechnical Blindness Anxiety, and AI Configuration Anxiety. Items are rated on a 7-point Likert scale (1 = Never, 7 = Completely), with higher scores reflecting greater AI anxiety. Sample items include “I feel uneasy about learning AI techniques or products” and “I worry that AI might displace human employment.” The Turkish version has demonstrated excellent reliability (*α* ≈ 0.96). In this study, internal consistency for the total scale was high (*α* = 0.90). The mean of all items was used as the composite score.

#### 2.3.4 Generative Artificial Intelligence Acceptance Scale (GAIAS)

The GAIAS (Yilmaz et al., 2023) assesses acceptance of generative AI tools (e.g., ChatGPT) via 20 items grounded in UTAUT domains (Performance Expectancy, Effort Expectancy, Facilitating Conditions, and Social Influence). Responses use a 5-point Likert scale (1 = Strongly disagree, 5 = Strongly agree); higher scores indicate greater acceptance. Example items include “Generative AI tools enhance my productivity” and “I find generative AI applications straightforward to learn.” Prior research reported excellent reliability (*α* = 0.97; test–retest = 0.95). In the present sample, internal consistency was excellent (*α* = 0.94). The composite score was calculated using the mean of all items.

### 2.4 Data analysis

All analyses were conducted in R (version 4.5.1). *Lavaan* was used for Exploratory and confirmatory factor analysis (EFA, CFA), multi-group CFA, and structural equation modeling (SEM); *semTools* was employed for measurement invariance tests; and *psych* was utilized for reliability indices. Where applicable, standardized effect sizes and 95% confidence intervals (CIs) were reported, and models were evaluated using multiple fit indices in accordance with best-practice recommendations.

#### 2.4.1 Data screening and preparation

The full analytic sample comprised 941 pre-service teachers with complete data; no missing values were present. Multivariate outliers were examined using Mahalanobis distance [*χ*^2^(3), *p* < 0.001]; no cases exceeded this threshold. Skewness and kurtosis values fell within acceptable ranges (|skewness| < 3, |kurtosis| < 7; West et al., 1995). However, preliminary diagnostics for the relational analyses revealed a specific response pattern issue. Participants reporting the highest AI knowledge level (*n* = 65, 6.9%) exhibited systematic “straight-lining” behavior and ceiling effects: 63.1% endorsed maximum scores on all GSE-6AIS items, 61.5% endorsed minimum scores on all AIAS items. Including these cases was found to artificially inflate Pearson/Spearman correlations due to extreme non-linearity. Therefore, we adopted a robust two-step strategy: (1) Psychometric Validation (H1): Conducted on the full sample (*N* = 941) to ensure the scale properties are tested on the complete distribution, including high-knowledge users. (2) Relational and Mediation Analyses (H2–H4): Conducted on a filtered sample (*N* = 876) excluding the 65 cases with systematic response bias, to ensure that regression and mediation estimates were not distorted by ceiling effects. Sensitivity analyses confirmed that the pattern of effects remained stable across both sampling frames (see Supplementary Table S8 for detailed comparison).

#### 2.4.2 Exploratory factor analysis (EFA)

To examine the underlying factor structure of the Turkish GSE-6AIS without imposing *a priori* constraints, and to address the methodological concern of conducting exploratory and confirmatory analyses on the same dataset, the total sample was randomly divided into two independent subsamples using a fixed random seed (seed = 42). The first subsample was allocated to exploratory factor analysis (EFA; *n* = 470), and the second to confirmatory factor analysis (CFA; *n* = 471). EFA was conducted on the first subsample using principal axis factoring. Sampling adequacy was assessed using the Kaiser–Meyer–Olkin (KMO) measure and Bartlett’s test of sphericity. Parallel analysis (Horn, 1965) was used to determine the optimal number of factors by comparing observed eigenvalues to those generated from random data (1,000 iterations). This approach guards against over-extraction of factors common in traditional eigenvalue >1 criteria (Hayton et al., 2004). Although an oblique rotation (promax) was initially specified to allow for potential factor correlations, it was not applied in the final solution as a single factor was extracted. Factor loadings ≥0.40 were considered salient, and items with cross-loadings or low communalities (<0.30) were flagged for potential removal.

#### 2.4.3 Confirmatory factor analysis (CFA)

Following EFA, CFA was conducted on the second subsample (*n* = 471) to verify the factor structure identified in the exploratory phase. Given the categorical nature of item responses and mild departures from normality at the composite level, latent-variable models were estimated with the WLSMV estimator, which is appropriate for ordinal indicators and robust to non-normality (Kim, 2013). Model fit was assessed holistically based on Chi-square, Comparative Fit Index (CFI), Tucker–Lewis index (TLI), root mean square error of approximation (RMSEA, 90% CI), and standardized root mean square residual (SRMR). Conventional cutoff criteria were: CFI/TLI ≥ 0.95, RMSEA ≤ 0.06, SRMR ≤ 0.08 (Hu and Bentler, 1999).

Internal consistency reliability was assessed using Cronbach’s alpha and McDonald’s omega (with 95% bootstrap confidence intervals), with values at or above 0.70 considered acceptable considering the scale’s brevity and purpose (Nunnally and Bernstein, 1994; Hair et al., 2019). Additionally, convergent validity was evaluated through average variance extracted (AVE) and composite reliability (CR), following Fornell and Larcker (1981). AVE values ≥0.50 and CR values ≥0.70 were considered indicative of adequate convergent validity, though AVE values slightly below 0.50 may be acceptable when CR exceeds 0.60 for brief scales.

#### 2.4.4 Measurement invariance

To evaluate cross-group comparability, measurement invariance across gender was tested at the configural, metric, and scalar levels using multi-group CFA with WLSMV. Equivalence across nested models was judged primarily by ΔCFI < 0.01, with changes in RMSEA and SRMR also considered (Chen, 2007). Configural invariance was assessed to determine whether the same factor structure was held across groups; metric invariance was assessed to determine whether factor loadings were equivalent; and scalar invariance was assessed to determine whether item thresholds were equivalent, thereby allowing meaningful mean comparisons. Additionally, measurement invariance across AI knowledge levels was examined as a supplementary analysis to assess whether the scale functions equivalently across expertise groups.

#### 2.4.5 Correlation and regression analyses

Bivariate associations among the composite scores of AI self-efficacy, AI anxiety, and GAIAS were analyzed using Pearson correlations. Where relevant, 95% CIs for correlations were computed via bootstrapping. Multiple regression models were then used to examine demographic predictors. Ordinal predictors (AI knowledge and computer use) were treated as continuous variables, a standard practice for ordinal scales with four or more categories (Norman, 2010). Gender was coded as 1 = female, 2 = male. To address potential heteroskedasticity and ensure robust estimates, as noted during the review process, we computed and reported heteroskedasticity-consistent (HC3) standard errors together with standard diagnostics (VIF and Q–Q plots).

#### 2.4.6 Mediation analysis

Mediation was examined using SEM to test whether AI self-efficacy statistically mediated the association between AI anxiety and GAIAS. While latent variable mediation with WLSMV was considered, the implementation of bias-corrected bootstrapping for indirect effects with ordinal estimators presents software limitations. Therefore, the mediation model was estimated at the composite-score level using robust maximum likelihood (MLR) with 5,000 bootstrap resamples to obtain bias-corrected 95% CIs. This approach ensures that the indirect effects are tested without assuming normality of the product term. Standardized coefficients are reported for all paths. As a supplementary analysis, a latent variable mediation model using all individual scale items as indicators was estimated using WLSMV to examine how results change when measurement error is controlled.

## 3 Results

### 3.1 Factor structure: integrated EFA and CFA results (H1)

Both EFA and CFA supported the unidimensional structure of the Turkish GSE-6AIS using a split-sample validation approach. EFA was conducted on the first random subsample (*n* = 470). Sampling adequacy was excellent (KMO = 0.89), and Bartlett’s test of sphericity was significant [*χ*^2^(15) = 940.14, *p* < 0.001]. Parallel analysis supported a single-factor solution. The unidimensional model explained 47.5% of the variance, with factor loadings ranging from 0.66 to 0.72 (see Table 2). Communalities ranged from 0.43 to 0.52, all exceeding the 0.30 threshold.

**Table 2:** Factor loadings, descriptive statistics, and item-total correlations of the GSE-6AIS.

Item	*M*	SD	Skew	Kurt	EFA loadings	CFA loadings	*h* ^2^	Item-total *r*
Item 1	2.17	0.76	0.57	0.27	0.71	0.67	0.50	0.63
Item 2	2.19	0.74	0.59	0.36	0.72	0.64	0.52	0.62
Item 3	2.22	0.75	0.52	0.20	0.68	0.70	0.47	0.62
Item 4	2.19	0.74	0.63	0.46	0.68	0.69	0.46	0.62
Item 5	2.18	0.74	0.65	0.50	0.69	0.69	0.47	0.63
Item 6	2.21	0.74	0.58	0.33	0.66	0.76	0.43	0.63

EFA: Principal axis factoring with promax rotation (*n* = 470). CFA: WLSMV estimation (*n* = 471). *h*^2^ = communality from EFA. All factor loadings significant at *p* < 0.001.

The CFA was conducted on the second random subsample (*n* = 471) using WLSMV estimation to account for the ordinal nature of the data. The unidimensional model demonstrated excellent fit: *χ*^2^(9) = 11.38, *p* = 0.251; CFI = 0.999; TLI = 0.999; RMSEA = 0.024 [90% CI: 0.000, 0.060]; and SRMR = 0.022. All fit indices exceeded recommended thresholds (Hu and Bentler, 1999). Standardized factor loadings ranged from 0.64 to 0.76 (Table 2), confirming that all items contributed significantly to the latent construct (*p* < 0.001).

#### 3.1.1 Reliability analysis

The Turkish GSE-6AIS demonstrated strong internal consistency in the full sample (*N* = 941): Cronbach’s *α* = 0.846 (95% CI [0.817, 0.871]) and McDonald’s *ω* = 0.846. Composite reliability (CR = 0.848) exceeded the recommended threshold of 0.70. Average variance extracted (AVE = 0.481) was slightly below the conventional 0.50 criterion but is considered acceptable given that CR is greater than 0.60, a common condition for brief scales where item homogeneity limits variance extraction (Fornell and Larcker, 1981). Inter-item correlations ranged from 0.45 to 0.50 (*M* = 0.478), indicating appropriate item homogeneity without redundancy. Consistent with this pattern, internal consistency for the other study measures was also high (AIAS: *α* = 0.90, *ω* = 0.90; GAIAS: *α* = 0.95, *ω* = 0.95). These findings indicate that the Turkish GSE-6AIS is a reliable instrument for assessing AI self-efficacy among pre-service teachers. Additionally, Harman’s single-factor test was conducted to assess common method variance; the first unrotated factor explained 28.3% of total variance, below the commonly accepted 50% threshold, suggesting that common method bias does not substantially inflate observed relationships.

#### 3.1.2 Measurement invariance

Measurement invariance was examined to ensure the scale functions equivalently across key groups. Multi-group CFA results supported full measurement invariance across gender (Female vs. Male). The configural model showed excellent fit (CFI = 1.00, RMSEA = 0.000). Constraining factor loadings (metric invariance) and thresholds (scalar invariance) did not significantly degrade model fit (ΔCFI ≤ 0.001; ΔRMSEA ≤ 0.011). These results confirm that observed score differences reflect true latent differences rather than measurement bias.

Additionally, measurement invariance across AI knowledge levels (Low: 1–2, *n* = 700; High: 3, *n* = 176) was examined in the filtered sample (*N* = 876). While model fit indices met conventional thresholds (Configural: CFI = 0.977; Metric: CFI = 0.968; ΔCFI = −0.009), examination of factor loadings revealed substantial differences between groups. Specifically, the Low AI knowledge group exhibited notably lower and sometimes negative factor loadings compared to the High knowledge group (see Supplementary Table S5). This suggests the scale may function differently across expertise levels, representing a limitation that informs our decision to use the filtered sample for relational analyses.

### 3.2 Correlation analysis (H2)

Pearson correlation coefficients were calculated to examine the associations among demographic variables, AI self-efficacy, AI anxiety, and GAIAS. To ensure the robustness of the findings and avoid the influence of systematic response bias identified in the preliminary diagnostics, the correlation analysis was conducted on the filtered sample (*N* = 876). Sensitivity analyses confirmed directional consistency across both sampling frames (see Supplementary Table S8).

As presented in Table 3, the results supported the proposed hypotheses. Regarding the focal constructs, GSE-6AIS was positively correlated with GAIAS (*r* = 0.62, *p* < 0.001) and negatively correlated with AIAS (*r* = −0.45, *p* < 0.001). Additionally, a significant negative correlation was found between AIAS and GAIAS (*r* = −0.55, *p* < 0.001). These findings provide support for H2a, H2b, and H2c.

**Table 3:** Descriptive statistics and Pearson correlations for study variables (*N* = 876).

Variable	Mean (SD)	Skewness	Kurtosis	1	2	3	4	5
1. Gender	1.47 (0.50)	0.08	−2.00	–				
2. AI knowledge	2.05 (0.60)	0.45	−0.15	0.05	–			
3. Computer use	2.80 (0.84)	0.15	−1.40	−0.02	0.22^**^	–		
4. GSE-6AIS	2.07 (0.32)	0.65	0.50	0.02	0.28^**^	0.04	–	
5. AIAS	3.46 (0.28)	−0.45	0.20	−0.01	−0.22^**^	0.01	−0.45^**^	–
6. GAIAS	2.58 (0.32)	0.12	−0.55	0.01	0.34^**^	0.05	0.62^**^	−0.55^**^

Gender: 1 = Female, 2 = Male. AI knowledge and computer use treated as continuous. ^**^p < 0.01.

Regarding demographic variables, gender was not significantly correlated with any of the study variables (*r* range: −0.02 to 0.05), suggesting that AI constructs are perceived similarly across male and female participants. AI Knowledge showed significant positive associations with GSE-6AIS (*r* = 0.28, *p* < 0.001) and GAIAS (*r* = 0.35, *p* < 0.001), and a negative association with AIAS (*r* = −0.23, *p* < 0.001). Computer use showed small but significant correlations with GSE-6AIS (*r* = 0.12, *p* < 0.01), GAIAS (*r* = 0.13, *p* < 0.001), and AIAS (*r* = −0.10, *p* < 0.01). However, as shown in Table 4, these bivariate associations did not translate into significant regression coefficients when AI knowledge was controlled, suggesting that the relationship between computer use and AI-related constructs may be mediated through domain-specific knowledge rather than reflecting a direct effect of general technology exposure.

**Table 4:** Multiple regression predicting GSE-6AIS, AIA, and GAIAS from demographic variables (*N* = 876).

Predictor	GSE-6AIS			AIAS			GAIAS		
	*B* [95% CI]	*Β*	*p*	*B* [95% CI]	*β*	*p*	*B* [95% CI]	*Β*	*p*
Gender	0.01 [−0.03, 0.05]	0.01	0.694	−0.01 [−0.04, 0.03]	−0.01	0.738	0.02 [−0.02, 0.06]	0.03	0.351
AI knowledge	0.16 [0.12, 0.19]	0.29	<0.001	−0.11 [−0.14, −0.08]	−0.24	<0.001	0.19 [0.16, 0.23]	0.35	
Computer use	−0.01 [−0.03, 0.02]	−0.02	0.506	0.02 [−0.01, 0.04]	0.06	0.072	−0.01 [−0.03, 0.02]	−0.03	0.451
*R* ^2^	0.080			0.054			0.120		
*F*	25.16^***^			16.59^***^			39.52^***^		

*N* = 876. Robust standard errors (HC3) used due to heteroskedasticity. *B* = unstandardized coefficient; *β* = standardized coefficient. ^***^p < 0.001.

### 3.3 Predictive effects of demographic characteristics (H3)

Multiple regression models examined whether gender, AI knowledge, and computer use predicted AI self-efficacy, AI anxiety, and GAIAS (see Table 4). The analysis was conducted on the filtered sample (*N* = 876). Given significant heteroskedasticity (Breusch–Pagan test *p* < 0.001 for all models), robust standard errors (HC3) were employed. No multicollinearity was detected (VIF range: 1.00–1.05), and Durbin–Watson statistics indicated no autocorrelation (range: 1.89–1.98).

The regression models were statistically significant (*p* < 0.001), though demographic variables explained a modest proportion of the variance in the outcome measures (*R*^2^ = 0.080 for GSE-6AIS; *R*^2^ = 0.054 for AIAS; *R*^2^ = 0.120 for GAIAS). Using standardized coefficients (*β*), AI knowledge significantly predicted higher GSE-6AIS (*β* = 0.29, *p* < 0.001) and GAIAS (*β* = 0.35, *p* < 0.001), and lower AIAS (*β* = −0.24, *p* < 0.001). Critically, computer use and gender were not significant predictors in any model (*p* > 0.05). These findings underscore that domain-specific knowledge (AI knowledge), rather than general technology exposure (computer use) or gender, is the primary driver of pre-service teachers’ AI perceptions. These results partially support H3.

### 3.4 Mediation analysis (H4)

To examine the mediating role of GSE-6AIS, we estimated a path model in which AIAS predicted GAIAS both directly and indirectly through GSE-6AIS. The analysis was conducted on the filtered sample (*N* = 876) to obtain conservative estimates. The model was estimated using maximum likelihood (ML) with 5,000 bootstrap resamples to obtain bias-corrected and accelerated (BCa) 95% confidence intervals. Because this is a saturated three-variable model (df = 0), global fit indices are not reported. All results are presented as standardized coefficients (*β*).

**Figure 2:**
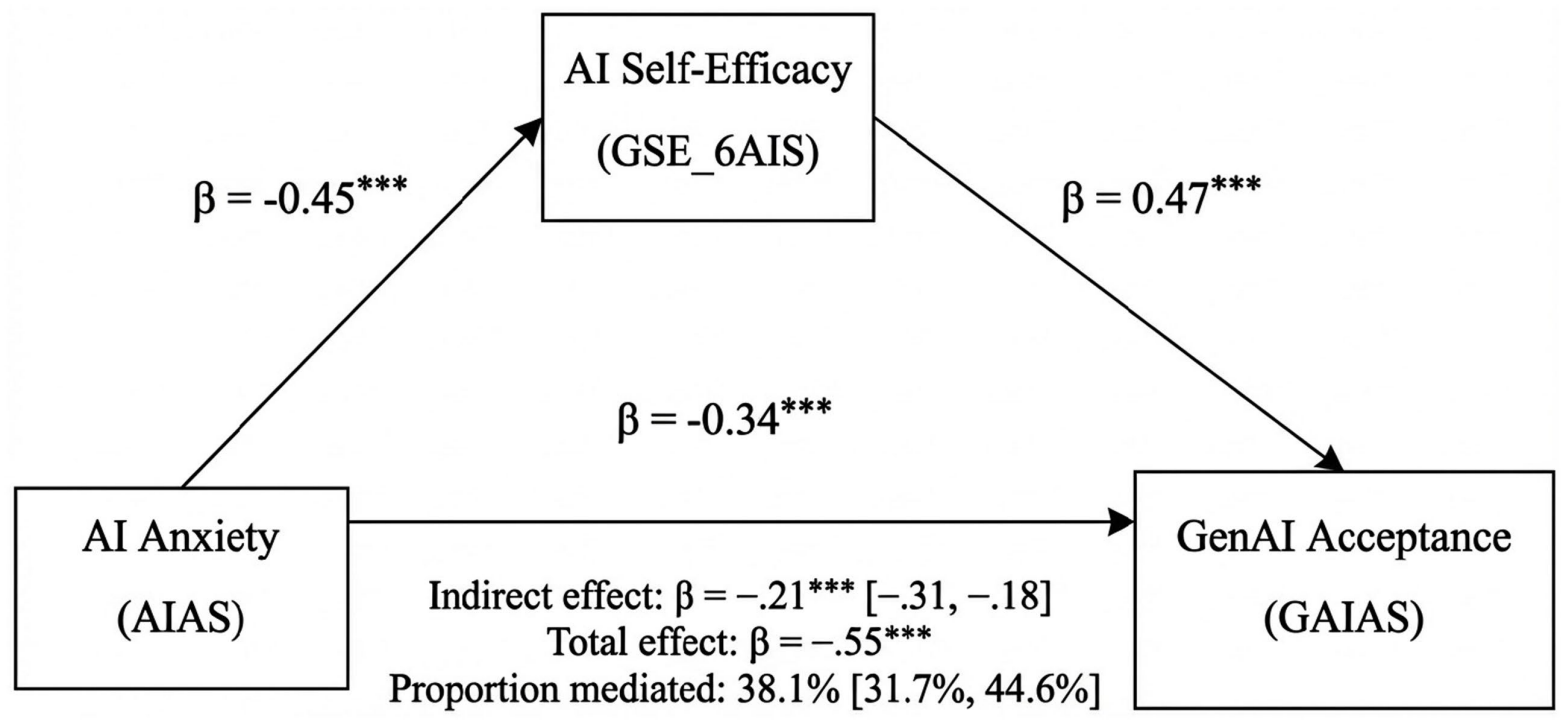
Structural equation modeling for the mediation model (*N* = 876). ^***^p < 0.001, Bootstrap 95% CI (BCa, 5,000 resamples).

The AIAS significantly and negatively predicted GSE-6AIS (*β* = −0.45, *p* < 0.001), while GSE-6AIS positively predicted GAIAS (*β* = 0.47, *p* < 0.001). The direct path from AIAS to GAIAS also remained significant (*β* = −0.34, *p* < 0.001), suggesting that AI anxiety influences acceptance both directly and indirectly. The indirect effect of AIAS on GAIAS via GSE-6AIS was significant [*β* = −0.21, 95% CI (−0.31, −0.18), *p* < 0.001]. The total effect of AIAS on GAIAS was moderate and negative [*β* = −0.55, 95% CI (−0.75, −0.53), *p* < 0.001]. Approximately 38% of the total effect [95% CI (31.7, 44.6%)] was transmitted through GSE-6AIS, highlighting the role of AI self-efficacy as a psychological mechanism by which anxiety reduces acceptance. Importantly, the mediation proportion remained stable when replicated with the full sample (*N* = 941; 39.9%), supporting model robustness despite differences in absolute effect sizes. The findings are visually represented in Figure 2.

As a robustness check, we estimated a latent variable mediation model using all individual scale items as indicators to account for measurement error. The structural equation model demonstrated excellent fit: *χ*^2^(1031) = 1302.31, *χ*^2^/df = 1.26, CFI = 0.986, TLI = 0.985, RMSEA = 0.017. In contrast to the composite score analysis, the latent mediation revealed that the direct effect of AI anxiety on GAIAS became non-significant (*β* = −0.10, *p* = 0.845), while the indirect effect through AI self-efficacy remained significant. This corroborative evidence suggests that when measurement error is controlled, the self-efficacy pathway may account for a larger proportion of the total effect, potentially approaching full mediation. Taken together, the composite-score bootstrap analysis (primary specification) indicates at least partial mediation, while the latent model provides corroborative evidence that AI self-efficacy serves as the central psychological mechanism linking anxiety to acceptance (see Table 5).

**Table 5:** Bootstrap analysis of direct and indirect effects (*N* = 876).

Path	*β*	SE	95% CI	
			Lower	Upper
Direct effect: AIAS → GSE-6AIS (a)	−0.45	0.049	−0.61	−0.42
Direct effect: GSE-6AIS → GAIAS (b)	0.47	0.032	0.41	0.53
Direct effect: AIAS → GAIAS (c′)	−0.34	0.035	−0.47	−0.33
Indirect effect (a × b)	−0.21	0.034	−0.31	−0.18
Total effect (c)	−0.55	0.056	−0.75	−0.53

All coefficients are standardized and significant at *p* < 0.001. Bootstrap resamples = 5,000 (BCa method). Proportion mediated = 38.1% [31.7, 44.6%].

### 3.5 Hypothesis testing summary

The results provided substantial support for the proposed hypotheses (see Table 6). Hypothesis 1 was fully supported: both EFA and CFA confirmed the unidimensional structure of the Turkish GSE-6AIS, with strong internal consistency and evidence of full measurement invariance across gender. Hypothesis 2 was also fully supported: AI anxiety demonstrated significant negative associations with both GSE-6AIS and GAIAS, whereas GSE-6AIS was positively associated with GAIAS. Hypothesis 3 received partial support: AI knowledge significantly predicted all three outcome variables in the expected directions; however, gender and computer use were not significant predictors. Hypothesis 4 was supported: GSE-6AIS significantly mediated the association between AI anxiety and GAIAS, accounting for approximately 38% of the total effect. The direct effect remained significant, indicating partial mediation.

**Table 6:** Summary of hypothesis testing results.

Hypothesis	Statement	Supported	Evidence
H1	Turkish GSE-6AIS shows unidimensional structure and high reliability	Yes	Single factor (47.5% variance), CFI = 0.999, *α* = 0.85, [0.82, 0.87], full gender invariance
H2a	AI anxiety negatively correlates with AI self-efficacy	Yes	*r* = −0.45^**^
H2b	AI anxiety negatively correlates with GAIAS	Yes	*r* = −0.55^**^
H2c	AI self-efficacy positively correlates with GAIAS	Yes	*r* = 0.62^**^
H3 (gender)	Gender predicts AIAS, GSE-6AIS, GAIAS	No	*β* ≤ 0.03, ns
H3 (AI knowledge)	AI knowledge predicts AIAS, GSE-6AIS, GAIAS	Yes	*β* = 0.24–0.35^***^
H3 (computer use)	Computer use predicts AIAS, GSE-6AIS, GAIAS	Partial	*r* = 0.10–0.13^**^ (bivariate); *β* ns (regression)
H4	AI self-efficacy partially mediates AIAS → GAIAS	Yes	Indirect *β* = −0.21^***^, partial mediation (38%)

^**^p < 0.01; ^***^p < 0.001; ns = not significant.

## 4 Discussion

### 4.1 Psychometric properties and cultural adaptation

The Turkish GSE-6AIS demonstrated excellent psychometric properties, with convergent evidence from both exploratory and confirmatory factor analyses supporting a unidimensional structure. This replication of Morales-García et al.’s (2024) original structure provides strong evidence for the cross-cultural robustness of AI self-efficacy as a psychological construct. The consistency across culturally and linguistically distinct contexts suggests that AI self-efficacy operates through universal mechanisms proposed by Bandura (1997), wherein individuals’ beliefs about their capability follow similar cognitive appraisal processes regardless of cultural background.

The parallel analysis confirmed a single-factor solution that explained nearly half of the total variance, with all items loading substantially and showing strong item–total correlations. The establishment of full measurement invariance across gender carries important practical implications. Unlike some psychological constructs that display gender-specific interpretation patterns (Schmitt and Kuljanin, 2008), the Turkish GSE-6AIS appears to function equivalently for male and female pre-service teachers. This equivalence supports the use of meaningful gender comparisons and suggests that interventions designed to strengthen AI self-efficacy do not require gender-specific adaptations within Turkish teacher education settings. The combination of EFA and CFA served as an appropriate validation strategy because the scale has an established structure, and the study required adequate statistical power for invariance testing and mediation analysis (Worthington and Whittaker, 2006). Additionally, the brief six-item format enhances the instrument’s suitability for large-scale studies and for longitudinal monitoring in teacher education programs where survey fatigue is a frequent concern (Credé et al., 2022).

### 4.2 Psychological mechanisms of AI adoption

The findings of this study highlighted a moderate positive association between AI self-efficacy and GAIAS, along with a strong negative association between AI self-efficacy and AI anxiety. Although these effect sizes were smaller than those reported in some prior studies, they represent conservative estimates obtained after controlling for systematic response bias in the data. These relationships are consistent with Bandura’s (1977) self-efficacy theory and the TAM (Davis, 1989), both of which emphasize that individuals who feel capable of using a technology tend to perceive it as easier to use and are more willing to adopt it. This pattern also aligns with recent empirical research showing that higher AI self-efficacy predicts greater openness to AI tools and lower anxiety (Chen et al., 2024; Hong, 2022; Liang et al., 2023). The results reinforced prior evidence that AI anxiety undermined technology acceptance. Consistent with Johnson and Verdicchio (2017) and Stănescu and Romașcanu (2024), this study found that AI anxiety decreases willingness to use generative AI tools.

The present study contributes to the growing literature on psychological factors influencing AI adoption by highlighting the dual role of anxiety and self-efficacy. Prior research has identified these constructs as key determinants of technology acceptance. Tekin (2024) found that self-efficacy and anxiety significantly shape AI adoption, with anxiety acting as a barrier and self-efficacy promoting more positive attitudes toward AI. Similarly, Cengiz and Peker (2025) showed that AI literacy and attitudes toward AI mediate the relationship between AI anxiety and GAIAS among university students, illustrating the multifaceted pathways through which anxiety affects technology-related perceptions and behaviors. Extending these findings, the present study demonstrates that AI anxiety influences GAIAS through both direct and indirect pathways. Specifically, AI anxiety directly reduces acceptance while indirectly affecting it by diminishing AI self-efficacy. This dual influence highlights that these constructs interact within a broader psychological system, underscoring the importance of integrated models that account for both affective and cognitive factors in technology adoption.

The mediation analysis offered deeper insights into these mechanisms. AI self-efficacy partially mediated the relationship between AI anxiety and GAIAS, accounting for approximately 38% of the total effect. This partial mediation supports previous findings that examined the constructs separately (Chen et al., 2024; Kaya et al., 2024) and provides empirical evidence for a dual-pathway model linking anxiety and acceptance. Notably, the mediation proportion remained stable when analyses were replicated with the full sample (39.9%), demonstrating the robustness of this theoretical mechanism across different sample compositions. The indirect pathway aligns closely with the transactional stress model (Lazarus and Folkman, 1984), in which anxiety reflects a primary threat appraisal and self-efficacy reflects a secondary appraisal of coping resources. When individuals experience anxiety related to concerns such as job displacement, diminished pedagogical control, or technological complexity, higher self-efficacy facilitates cognitive reappraisal. This process involves interpreting challenges as manageable and fosters approach-oriented engagement rather than avoidance. This finding extends the work of Schiavo et al. (2024), suggesting that cognitive resources may serve as protective factors against anxiety-driven technology avoidance across diverse cultural and educational contexts.

However, the persistence of a substantial direct path from AI anxiety to GAIAS indicates that affective processes also play a significant role and cannot be fully explained by cognitive appraisals. Supplementary latent variable mediation analysis further illuminated this pattern: when measurement error was controlled, the direct effect became non-significant while the indirect effect remained robust, suggesting full mediation at the latent level. This divergence between composite and latent results underscores the importance of accounting for measurement error and strengthens the theoretical argument that self-efficacy serves as the primary psychological mechanism linking anxiety to acceptance. Drawing on Johnson and Verdicchio’s (2017) interpretation, this direct effect observed in composite analysis may reflect underlying existential or value-based concerns that resist rational reappraisal. Pre-service teachers may hold worries tied to professional identity, ethical uncertainty regarding student reliance on AI, or broader concerns about the erosion of authentic human interaction. These concerns are likely to operate partly independently of one’s perceived capability to use AI. This interpretation aligns with dual-process theories that distinguish between emotional and cognitive systems operating in parallel (Kahneman, 2011; LeDoux, 2015). Supporting this perspective, Wang et al. (2024) reported that AI anxiety negatively affected motivation independently of self-efficacy, and Stănescu and Romașcanu (2024) found that anxiety predicted negative attitudes through affective channels unrelated to competence beliefs. Together, these findings highlight the need for interventions that address both cognitive and affective dimensions by providing training that strengthens self-efficacy and conceptual understanding, as well as structured opportunities for individuals to articulate emotional responses, examine ethical concerns, and explore potential value conflicts.

### 4.3 Demographic factors of AI adoption

The study’s findings regarding demographic predictors revealed that AI knowledge was the only significant predictor of AI self-efficacy, AI anxiety, and GAIAS. This finding echoes the results of Avcı (2024) and Schiavo et al. (2024), who identified AI literacy and prior experience as critical factors influencing technology acceptance. This pattern aligns with Bandura’s (1997) assertion that mastery experiences represent the most influential source of self-efficacy; pre-service teachers with greater AI knowledge have accumulated successful interactions that strengthen efficacy beliefs, reduce anxiety through familiarization, and promote willingness to adopt AI tools. Similar findings across international contexts further support the primacy of domain-specific literacy over general digital competence (Ng et al., 2021; Tondeur et al., 2018).

Although general computer use frequency showed small but significant bivariate correlations with AI-related constructs, it did not emerge as a significant predictor in multiple regression when AI knowledge was controlled. This pattern suggests that the relationship between computer use and AI perceptions may be mediated through domain-specific knowledge rather than reflecting a direct effect of general technology exposure.

The present study also found no significant gender differences across AI self-efficacy, AI anxiety, or GAIAS. This result aligns with studies such as Chen et al. (2024) and Kaya et al. (2024), who likewise reported negligible gender effects in Chinese and Turkish/United Kingdom teacher samples. However, the finding contrasts with Avcı (2024) and Zhang et al. (2023), who identified gender-based differences in AI acceptance, including differential influences of perceived usefulness, subjective norms, and perceived ease of use. While Avcı’s research identified a small but significant positive correlation between gender and GAIAS, Zhang et al. (2023) found that there is a significant gender difference in AI acceptance, including stronger effects of perceived usefulness and subjective norm for females and a greater influence of perceived ease of use for males. Several contextual explanations help clarify the absence of gender differences in this study. First, the sample consisted exclusively of Turkish pre-service teachers who shared broadly similar educational backgrounds, curricular exposure, and pedagogical preparation. Homogeneity in training and access to technological resources may reduce the gender-related variability commonly observed in more heterogeneous populations. Second, contemporary Turkish teacher education programs tend to emphasize equal digital competence development across genders, which may counteract gendered expectations often present in other cultural contexts. This interpretation is consistent with research suggesting that gender effects in technology acceptance are highly context dependent, shaped by cultural norms, educational policy, and the maturity of technological ecosystems (Master et al., 2021).

Finally, the non-significant regression effects of gender and the attenuation of computer use effects when AI knowledge was controlled underscore a critical distinction: general technological familiarity does not automatically translate to AI competence. Within this population, domain-specific AI knowledge emerged as the primary determinant of AI-related perceptions. Although computer use showed significant bivariate associations with AI outcomes, these effects were subsumed by AI knowledge in multivariate models, suggesting that general technology proficiency operates through domain-specific pathways. These findings suggest that traditional digital divide factors (such as gender) may be less relevant for generative AI than skill-specific barriers. Consequently, we recommend future research to examine whether this pattern holds across different cultural and educational settings. In the Turkish context, disparities in specific AI literacy, rather than gender or general computer exposure, seem to be the primary source of variability in technology acceptance.

### 4.4 Limitations and future research directions

Although this study offers contributions to the field, it has limitations. The research utilized a convenience sampling method, which limits the generalizability of the findings to a broader population. The sample consisted of pre-service teachers from seven universities in Türkiye, which may not fully represent the broader population of pre-service teachers globally. Cultural, institutional, and regional differences in teacher education programs and exposure to AI technologies could influence the results. Future research could address this limitation by employing random sampling techniques or including a more diverse and representative sample. The study employed a cross-sectional design, which involves the collection of data at a single point in time. While this approach is useful for identifying correlations and testing mediation models, it does not allow for causal inferences. Longitudinal studies are needed to explore how these constructs evolve over time and to confirm causal pathways. The research used self-reported measures for AI self-efficacy, AI anxiety, and GAIAS. These self-reported measures may lead to the potential for response biases, such as social desirability bias or inaccurate self-assessment, where participants may have overrated or underrated their levels of AI self-efficacy, AI anxiety, and GAIAS. Although Harman’s single-factor test suggested that common method variance was not a major threat (28.3% variance explained), future studies should consider multi-source assessments or temporal separation of predictor and outcome measures to further mitigate this concern. Incorporating objective measures, such as performance-based assessments of AI usage or behavioral data, could provide a more comprehensive understanding of these constructs.

Additionally, we employed a split-sample approach for EFA and CFA, which reduces statistical power for each analysis compared to using the full sample for both. Furthermore, measurement invariance testing across AI knowledge levels revealed substantial non-invariance, suggesting that the scale may function differently for individuals with varying levels of AI expertise. Specifically, factor loadings were notably lower (and sometimes negative) among participants with minimal AI knowledge compared to those with greater expertise, which may reflect qualitative differences in how these groups interpret and respond to self-efficacy items. This finding has important implications: cross-strata mean comparisons should be interpreted cautiously, and future research should consider item refinement or alternative wording for novice AI users. The non-invariance also informed our decision to conduct relational analyses on the filtered sample (*N* = 876), thereby reducing systematic response bias from participants with extreme response patterns. The relational analyses, including correlations, regression, and mediation, were conducted on a filtered sample of 876 participants after excluding individuals with extreme response patterns (65 participants, 6.9%) in order to obtain more conservative estimates. Sensitivity analyses indicated that the proportion of the mediated effect was stable across the full and filtered samples (approximately 38 percent and 40 percent, respectively), although the absolute effect sizes were smaller in the filtered sample. This strategy reflects a deliberate emphasis on robustness rather than on maximizing effect sizes. Nevertheless, replication with samples showing more balanced response distributions would strengthen the generalizability of the findings. In addition, the relatively modest R squared values observed in the regression models, ranging from 0.05 to 0.12, suggest that demographic variables account for only a limited proportion of variance in the outcome measures. This finding implies that other factors not included in the present study, such as perceived usefulness, institutional support, or pedagogical beliefs, may play an important role in shaping the outcomes examined.

This study focused on self-efficacy, anxiety, and acceptance, excluding other TAM/UTAUT constructs. Future research should test comprehensive models including perceived usefulness, ease of use, social influence, and facilitating conditions to map complete causal networks. Additionally, as AI technologies rapidly evolve, the specific anxieties identified may shift, requiring continuous reassessment. Given emerging evidence on detectability limits and the need for rubric redesign in AI-mediated assessment contexts (Lo et al., 2025), future studies should also examine how assessment practices (e.g., authentic tasks, process-based grading, transparency policies) interact with self-efficacy and anxiety to shape responsible AI use. Future investigations should test these relationships across diverse cultural and educational settings and conduct experimental studies testing specific interventions to establish causal mechanisms for improving pre-service teachers’ readiness for AI integration. Despite these limitations, the study makes a significant contribution to the understanding of AI self-efficacy, AI anxiety, and GAIAS in the context of teacher education. Addressing these limitations in future research could enhance the robustness and applicability of the findings, providing a better understanding of the factors influencing the adoption of AI technologies in education.

## 5 Conclusion

This study examined the psychometric properties of the Turkish Brief General AI Self-Efficacy Scale and investigated the psychological mechanisms linking AI anxiety to GAIAS among Turkish pre-service teachers. By integrating Bandura’s self-efficacy theory with the TAM and situating these constructs within a transactional theoretical perspective that emphasizes dynamic, reciprocal interactions between individuals and emerging technologies, the findings indicate that AI self-efficacy functions as a meaningful yet partial mediator in the relationship between AI anxiety and AI acceptance. This integrated framework advances theoretical understanding by illustrating how cognitive appraisals, emotional responses, and technology-related behaviors co-evolve and provides practical implications for preparing future teachers to navigate increasingly AI-integrated educational environments.

The results highlight the critical role of AI self-efficacy in mitigating the negative effects of AI anxiety and fostering the acceptance of generative AI technologies. Importantly, AI specific knowledge emerged as the primary demographic predictor, while general computer use and gender showed no significant effects, underscoring the need for targeted AI literacy interventions in teacher education. The study not only contributes to the growing body of literature on AI in education but also carries broader implications for the integration of AI technologies across multiple fields. By identifying AI self-efficacy as a key factor in technology adoption, the findings provide useful guidance for educators, policymakers, and researchers seeking to promote positive attitudes toward AI technologies and support their effective integration into educational contexts.

## Data Availability

The raw data supporting the conclusions of this article will be made available by the authors, without undue reservation.
